# Regulatory T cells: A potential weapon to combat COVID‐19?

**DOI:** 10.1002/mco2.12

**Published:** 2020-08-06

**Authors:** Yu Liu, Guangying Qi, Joseph A. Bellanti, René Moser, Bernhard Ryffel, Song Guo Zheng

**Affiliations:** ^1^ Department of Clinical Immunology Sun Yat‐sen University Third Affiliated Hospital Guangzhou P. R. China; ^2^ Guangxi Key Laboratory of Tumor Immunology and Microenvironmental Regulation Guilin Medical University Guilin P. R. China; ^3^ Department of Pediatrics and Microbiology‐Immunology Georgetown University Medical Center Washington District of Columbia; ^4^ Institute for Biopharmaceutical Research Matzingen Switzerland; ^5^ Experimental and Molecular Immunology and Neurogenetics (INEM) UMR 7355 INEM CNRS‐University of Orleans Orleans France; ^6^ Department of Internal Medicine Ohio State University College of Medicine and Wexner Medical Center, Medical Center Columbus Ohio

**Keywords:** 2019 novel coronavirus, pathology, pneumonia, SRAS‐CoV‐2, Tregs

## Abstract

Since the end of December 2019, a novel coronavirus SARS‐CoV‐2 began to spread, an infection disease termed COVID‐19. The virus has spread throughout the world in a short period of time, resulting in a pandemic. The number of reported cases in global reached 5 695 596 including 352 460 deaths, as of May 27, 2020. Due to the lack of effective treatment options for COVID‐19, various strategies are being tested. Recently, pathologic studies conducted by two teams in China revealed immunopathologic abnormalities in lung tissue. These results have implications for immunotherapy that could offer a novel therapy strategy for combating lethal viral pneumonia. This review discusses the clinical and pathological features of COVID‐19, the roles of immune cells in pathological processes, and the possible avenues for induction of immunosuppressive T regulatory cells attenuating lung inflammation due to viral infection. It is our hope that these proposals may both be helpful in understanding the novel features of SARS‐CoV‐2 pneumonia as well as providing new immunological strategies for treating the severe sequelae of disease manifestations seen in people infected with SARS‐CoV‐2.

## Graphical abstract

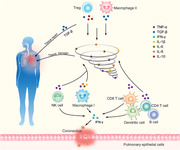

**Abbreviations**
ALI: acute lung injury
DAC: 5‐aza‐2′‐deoxycytidine
SARS‐CoV: severe acute respiratory syndrome coronavirus
Tregs: Regulatory immune cells

## 1 INTRODUCTION

During last two decades, a total of three times the coronavirus of animal origin infected human populations. For the first time in 2002, the severe acute respiratory syndrome coronavirus (SARS‐CoV) has outbroken; in 2012, the Middle East respiratory syndrome coronavirus (MERS‐CoV) outbroken for the second time; recently, in December 2019, a new coronavirus SARS‐CoV‐2 (also known as 2019‐nCoV) was isolated in human and has been responsible for outbreaks of infectious pneumonia and severe respiratory disease. Compared to other viruses, it is spreading rapidly and has reached global areas in all parts of Asia, Europe, North America, and South America in more than 180 countries in less than 4 months. As of May 27, 2020, there have more than 5 695 596 reported cases, with a mortality rate of 6.2% (https://promedmail.org/). The continuing epidemic threat of infection caused by this highly risk virus to global health has been considered a major concern by the Public Health of Emergency of International Concern (PHEIC), World Health Organization (WHO) on January 30, 2020.

Although SARS‐CoV‐2 positive patients initially present with loss of taste and fever with or without respiratory symptoms, many infected people develop various degrees of pulmonary inflammation demonstrable by chest Computed Tomography imaging.^1^, ^2^ Although the vast majority of patients present subclinical infection with mild form of illness, about 15‐20% of the patients do develop severe disease and critical condition requiring oxygenation treatment.^3^ The severely infected people have a high mortality rate; on average it reaches 6.3% and in some areas it is as high as 15.5%. The high lethal rate is associated with many factors, including medical resources of local and physical state of the infected person, such as obesity, underlying pre‐existent diseases (heart disease, diabetes), as well as older age. There is a crucial need for more intensive research of immunopathological mechanisms correlated with clinical onset and progression to understand pathogenesis, effective clinical practice, and novel therapeutic approaches.

## 2 OBSERVATIONS

### 2.1 Pathological changes in SARS‐CoV‐2 infections

#### 2.1.1 Early pathological changes in SARS‐CoV‐2‐infected pneumonia

The pathology data of COVID‐19 could be obtained by autopsies. Xiao and colleagues reported two lung cancer patients who were later diagnosed as COVID‐19 infected.^4^ When the superimposed infections were not recognized, lung tumors of the two patients were obtained by surgeries.^4^

The surgical specimens were presumed at the early phase of SARS‐CoV‐2 infection. The histopathology of surgical specimens showed inflammatory infiltrate and pneumocyte hyperplasia in the absence of obvious hyaline membrane formation, squamous metaplasia, and tissue remodeling consistent with proliferative and exudative features of acute lung injury (ALI).^4^ However, the mechanisms underlying pathogenesis of COVID‐19 are so far not fully understood.

#### 2.1.2 Later pathological changes in SARS‐CoV‐2‐infected pneumonia

**FIGURE 1:**
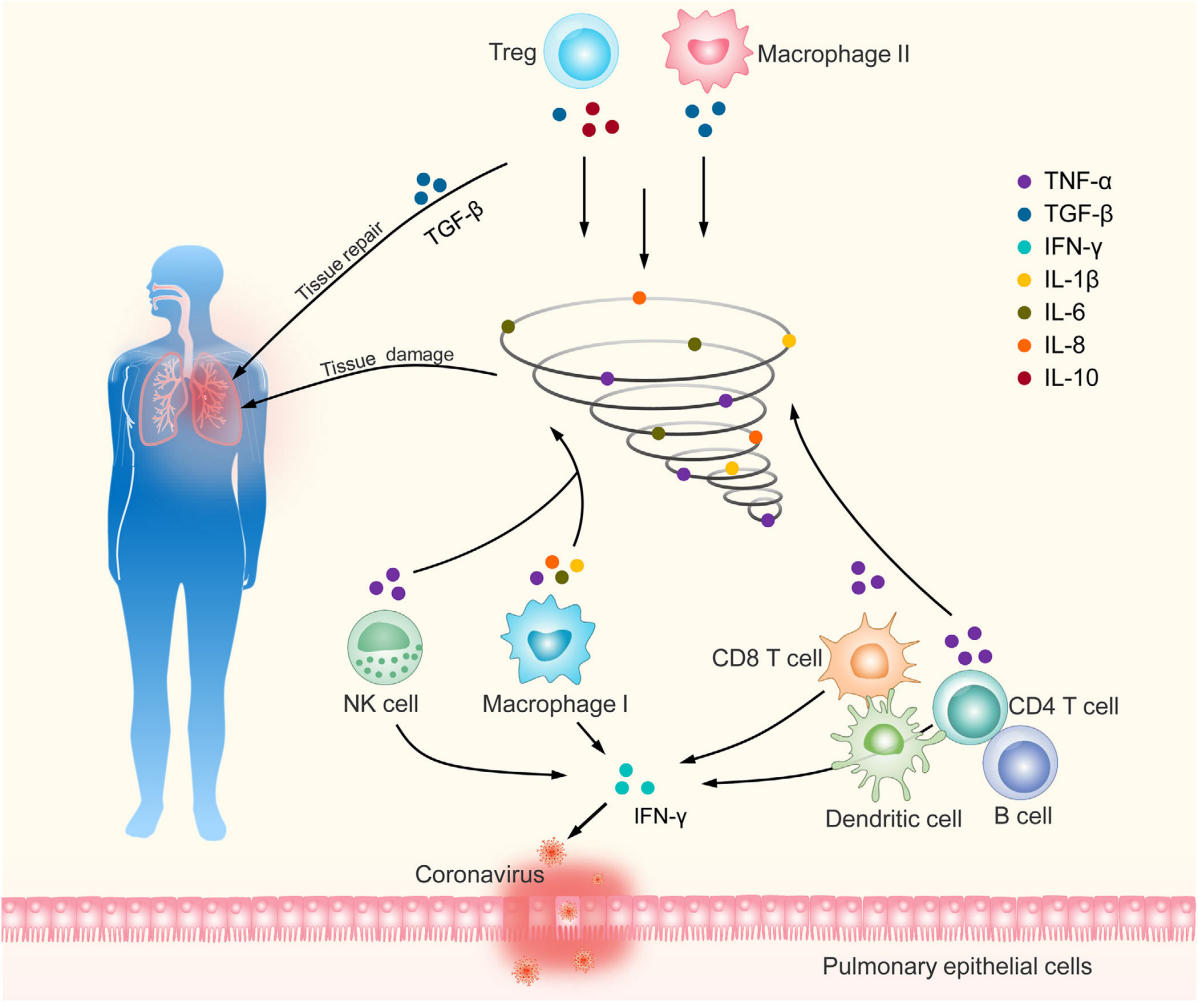
Immune response in lung tissues after coronavirus infection. When lung tissue is infected with coronavirus, NK cells, macrophages, and antigen‐presenting cells are recruited to the tissue to produce inflammatory factors and then active CD8 and CD4 T cells for combat coronavirus. When the infection is serious, these inflammatory factors will form inflammatory cytokines storm to injury the tissue. TNF‐α, IL‐1β, IL‐6, and IL‐8 are considered the main components of cytokine‐storm. TNF‐α would be produced by NK cells, macrophages, and activated CD4 and CD8 T cells. IL‐1β and IL‐8 are secreted by macrophages. IL‐6 could be produced by inflammatory macrophages. At the same time, T cells also secrete IFN‐γ to fight the coronavirus. On the opposite side, Treg and type II macrophage could secret IL‐10 and TGF‐β, which then reduce inflammatory response. In addition, TGF‐β participates in the tissue repair process

Xu et al^5^ were the first to report the pathological characteristics of a person who died from COVID‐19 by examining biopsy samples obtained at autopsy. The patient was a 50‐year‐old man whose chest showed many patchy shadows in right and left lungs by X‐ray.^5^ The histopathology of both lungs exhibited diffuse alveolar injury with fibromyxoid secretions.^5^ In addition to this, both lung tissues revealed desquamation of pneumocytes and hyaline membrane formation. The report pointed out that in the intra‐alveolar spaces of autopsy tissues emerged multinucleated syncytial cells with amphiphilic granular cytoplasm and enlarged pneumocytes, a characteristic of viral cytopathic‐like changes. In short, multinucleated giant cells and inflammatory cells were prominently observed within the interspace in the absence of any significant neutrophil infiltration in tissues. The report also demonstrated that the number of CD4 T cells and CD8 T cells were substantially decreased, whereas Th17 and CD8 cytotoxic T cells were significantly grown in the peripheral blood.^5^ Wan et al data also showed decreasing level of NK cells CD8^+^ T cells, B cells, and CD4^+^ T cells, and elevated levels of serum IL‐10 and IL‐6 in peripheral blood lymphocyte of SARS‐CoV‐2‐infected patients (unpublished). Another report by Huang et al^6^ showed that compared to healthy subjects, the initial levels of plasma IL‐10, IL‐8, IL‐1Rα, IL‐7, IL‐1β, IL‐9, basic FGF, GM‐CSF, G‐CSF, IP10, MIP1A, MIP1B, MCP1, IFN‐γ, TNF‐α, PDGF, and VEGF were higher in SARS‐CoV‐2‐infected patients than controls.^6^ Moreover, higher plasma levels of IL‐10, IL‐7, IL‐2, G‐SCF, MIP1A, MCP1, IP10, and TNF‐α were also observed in more severely affected patients assigned to an ICU than that non‐ICU patients.^6^ Such a mechanism was suggested by Huang et al^7^ in a study of SARS patients in which 14 chemokines or cytokines were analyzed on 88 RT‐PCR‐confirmed SARS‐CoV patients. The report showed that although TNF‐α, IL‐10, TNFRI, IL‐2, IL‐13, or IL‐4 levels were normal, the levels of IL‐18, IL‐8, IL‐6, IP‐10, IFN‐γ, MIG, TGF‐β, and MCP‐1 were highly increased in serum of the early phase of Taiwan SARS‐CoV patients. The authors concluded that an IL‐6‐, IFN‐γ‐, and IL‐8‐related inflammatory storm was excited after SARS‐CoV infection, and this inflammatory storm might cause immunopathological damage in SARS‐CoV patients. Therefore, it is reasonable to conclude that the clinical deterioration of patients infected with SARS‐CoV‐2 is perhaps due to increased systemic cytokine levels known as cytokine storm. The main changes in the microenvironment of infected pulmonary tissue include accumulating lots of NK cells, macrophages (mainly be type I macrophage), B cells, and dendritic cells, which can recruit and contribute to the activation of CD8 T and CD4 T cells (Figure 1). These cells will directly affect or secret above‐mentioned inflammatory factors to eliminate coronavirus but also cause lung inflammation and injury. At the same time, amount of natural regulatory T cell (nTreg) and induced regulatory T cell (iTreg) will be chelated to infected pulmonary tissue to inhibit excessive inflammation and repair the tissue (Figure 1). When the infection is serious, a bunch of inflammatory factors will form inflammatory storm and the pulmonary tissue damage, and the function of Treg cells will be then reduced or diminished.

#### 2.1.3 Treatment of SARS‐CoV‐2 pneumonia

Until now, no specific antiviral approach is validated to cure the patients of SARS‐CoV‐2 infection except for fine patient care. Currently, the most effective method to this infectious pneumonia is to block the origin of infection. Second is to take personal protection measures and provide early diagnosis and treatment for affected people.^8^, ^9^ Use of an antiviral therapeutic regimen consisting of an anti‐HIV drug combination of ritonavir and lopinavir together with intranasal nebulized IFN‐α at appropriate drug dosages was suggested by China's National Health Commission, and the guidance is dependent on and determined by disease severity.^10^, ^11^ Antibacterial agents are not only ineffective, but importantly, often cause serious acute respiratory distress syndrome.^7^ There are more than 200 clinical trials in SARS‐CoV‐2 patients with antivirus agents in China, and none has demonstrated any significantly therapeutic effect on patients with COVID‐19. Hence, an effective treatment strategy for COVID‐19 is urgent needed.

### 2.2 Use of regulatory T cells: A possibility of immunotherapy for SARS‐CoV‐2 patients

#### 2.2.1 Main characterization and function of Tregs

We propose here that application of T regulatory cells (Treg) may help prevent the disease transition from mild to severe and even to treat severe patients. It has been known that human immunological homeostasis is maintained by a group of immunosuppressive cells such as regulatory immune cells (Tregs).^12^, ^13^, ^14^ Tregs can inhibit multiple immune cells, such as CD8^+^, CD4^+^ T cells, monocytes, NK cells, as well as B cells controlling undesired immune response at different stages of transplant, allergy, and autoimmunity rejection.^15^, ^16^, ^17^, ^18^ Tregs exert this inhibitory function mainly by the secretion of cytokines TGF‐β and IL‐10. Tregs can interfer with CD8 T cell and CD4 T cell proliferation and survival through competing IL‐2, and inhibit antigen‐presenting cell maturation and eliminate effector cells through secreting cytokines.^17^, ^19^, ^20^ Most of Tregs are CD4^+^ T cells that highly express the IL‐2 receptor alpha chain (CD25), as well as express low levels of IL‐7 receptor (CD127) in humans.^21^ Importantly, the transcription factor Foxp3 is considered to be an important factor in Treg function and development, and is also recognized to be a specific marker for identifying and defining Treg from other immune cell subsets.^22^, ^23^ The subgroup of Treg CD4^+^CD25^high^CD127^low/−^FoxP3^+^ has been considered the archetypical Treg subset that could control the most basic aspect of suppressor cell‐mediated immune balance and tolerance in human.^24^, ^25^, ^26^ Tregs can be produced in thymus and periphery, which derive from thymus called natural Treg cells (nTregs), other of nonthymic‐origin called induced Treg cells (iTregs).^27^, ^28^, ^29^ In addition, by anti‐CD3/CD28‐coated beads can be combined with appropriate dose of cytokines IL‐2 and TGF‐β to induce iTregs in vitro from naïve T‐cells.^30^ These in vitro induced Treg cells share most functional and phenotypic characteristics with nTregs.^30^, ^31^, ^32^ In the past decade, people have made great efforts to understand and develop Tregs for treatment. Treg immunotherapy has become a legitimate quest for a clinical entity that would selectively block unwanted autoimmune and immune responses and, in some cases, a therapeutic regimen that could lead to the induction of tolerance.^33^, ^34^, ^35^

#### 2.2.2 Application of Treg in viral pneumonia

The immunopathology of viral pneumonia reflects a complex interaction between direct effects of the virus itself but also the response(s) of the adaptive and innate immune systems. Studies conducted in the past few decades have suggested that in most cases, but not in all, the extent and type of lung injury is dictated by immune response to the host respiratory viral infection, instead of the direct impact of viral replication.^36^

Several studies have shown that Tregs play an important role in controlling viral pneumonia and viral‐induced ALI.^13^, ^19^, ^37^, ^38^ In a mouse model study by Fulton et al,^38^ evaluating the balance between virus clearance and immunopathology, Foxp3^+^CD4^+^ regulatory T cells were shown to accumulate in mediastinal lymph nodes and the lungs of infected animals and to reduce immunopathology by regulating the responses of CD8 effector T cell during infection of respiratory syncytial virus.^38^ In a similar mouse study of ALI induced by LPS by Alessio et al,^13^ Tregs were significantly accumulated in the alveolar spaces. These results were compared to the findings of two patients with ALI who were also shown to have increased numbers of Tregs in their alveolar spaces and demonstrated that Tregs play an essential role in alleviating lung injury. Transferring Tregs to Rag‐1^−/−^ mice with ALI significantly improved survival of infected mice, in contrast to the effects of CD4‐depleted spleen cells or saline control infusion or CD4^+^CD25^−^ cells.^13^ In mediating these effects, TGF‐β was needed to realize resolution of ALI mediated by Tregs. In the absence of Tregs, the pro‐inflammatory responses induced by LPS were durable, and apoptosis of neutrophil was decreased.^13^ Another study by Antunes et al^37^ revealed similar results and demonstrated that transferring CD4^+^CD25^+^CD127^dim^ Tregs to Rag‐1^−/−^ mice infected by influenza A virus, that causes injury to the respiratory organs, led to significant delay in extended survival time after infection.^37^ In contrast, transferring CD4^+^CD25^+^CD127^dim^ Tregs could not change the replication rate of virus in the lungs of Rag‐1^−/−^ mice. In summary, Tregs therapy may provide potential opportunities for clinical intervention to rebuild immune tolerance in viral pneumonia.

#### 2.2.3 Molecular mechanism of Treg involved to viral pneumonia

As aforementioned, Treg cells act against to viral pneumonia indirectly by suppressing the immune response. It is likely that Treg cells use multiple mechanisms of suppression including secreted proteins and cell surface molecules. There are three secreted proteins that are highly identified with suppression of Tregs, including TGF‐β, IL‐10, and IL‐35.^38^ IL‐10 and TGF‐β, anti‐inflammatory cytokines, could suppress the activity of NK cells, macrophage, cytotoxic CD8 T cells, and T helper cells 1 to reduce inflammatory storm caused by those during virus infection (Figure 1). IL‐35 belongs to the family of IL‐12 cytokines and suppresses T‐cell proliferation through IL‐12Rβ2 and gp130 signaling.^39^

Cell surface molecules were proposed as a key mediator of Treg cell–mediated suppression. TIGIT and CTLA‐4, at high level on Tregs, were also implicated in suppression activity of Treg. The suppressive function of CTLA‐4‐deficient Tregs crippled as they are unable to downregulate costimulatory signal CD80 and CD86 via transendocytosis.^38^ TIGIT could induce secretion of TGF‐β and IL‐10 by dendritic cells.^38^ Other cell surface molecules such as CD73, CD39, LAG‐3, GITR, and PD‐1 also participate in suppression activity of Treg.^38^ CD73 and CD39 are two ectoenzymes and take part in suppressive function by facilitating the extrusion of cAMP and elaboration of adenosine.^38^ LAG‐3 negatively regulates T‐cell homeostasis and the expansion of activated T cells is considered necessary for maximal suppressive function of Treg cells.^40^ GITR, also termed TNFRSF18, is highly expressed in Treg cells. The inhibition activities of CD25^+^CD4^+^ T can be eliminated by stimulating GITR via indoleamine 2,3‐dioxygenase induced noncanonical NF‐κB–dependent signal pathway.^41^, ^42^ One study has shown that PD‐1 negatively regulated Tregs suppressive activity by limiting STAT‐5 phosphorylation.^43^

In addition to inhibition of inflammatory responses following virus infection, Tregs could promote tissue repair by expression of amphiregulin, which does not depend on the immunosuppressive activity of Treg.^39^ Taken together, these biological characteristics suggest that Treg cells may have multiple antivirus protection mechanisms.

#### 2.2.4 Therapeutic opportunities for Treg in SARS‐CoV‐2

As described above, Treg therapy may be one strategy for the treatment of this novel SARS‐CoV‐2 infection—COVID‐19. Rigorously designed clinical trials and detailed Treg renovation planning that utilize the latest scientific technology of Treg biology will be used to harness the carcinogenic potential of Treg immunotherapy. We suggest consideration of four possible lines of scientific exploration for Treg immunotherapy:

(1) Expansion and infusion of autologous CD4^+^CD25^high^CD127^low^ cells isolated from patients: One potential weakness of this approach is a temporal concern since this cell population requires 2–3 weeks for ex vivo expansion to achieve sufficiently quantities of cells for clinical use.^44^
(2) Use of induced CD4^+^ Tregs that are differentiated from naïve CD4^+^ T cells in vitro: Previous studies have demonstrated that the combination of TGF‐β and *all‐trans* retinoic acid has the capacity to develop human iTreg cells.^45^ This approach can be achieved in 1 week, which should allow the production of sufficient quantities of Treg cells to conduct clinical trials. Moreover, the addition of *all‐trans* retinoic acid during amplification greatly enhances the stability of iTregs.^46^, ^47^
(3) Use of antigen‐specific Tregs: The inclusion of antigen‐specific Tregs should have the added advantage of reducing the probability of off‐target immunosuppression. The results of studies performed in mouse models that we and other groups have used discovered that antigen‐specific Tregs have a stronger and more efficient function in controlling unwanted immune responses.^48^, ^49^ This approach would reduce the cell numbers and costs of the manufacturing process.
(4) Combination of Tregs and suppressive drugs. TNF‐α, IFN‐γ, and IL‐6 are cytokines known to cause tissue damage. The ability of TNF‐α and IL‐6 antagonists to reduce inflammation, induce an increase in the level of Treg, and promote Tregs development in vivo by combining TGF‐β with low dose of IL‐2 has led to their approval for treating autoimmune diseases.^50^ One study has shown that combining mutant IL‐2Fc plus IL‐15Fc and rapamycin could eliminate T effectors and promote Treg development.^51^ Additionally, another study has also shown that DNA methyltransferase inhibitor 5‐aza‐2′‐deoxycytidine could enhance Foxp3 expression, activation state, suppressive phenotype, and proliferative capacity to accelerate repair of experimental lung injury.^52^

As described previously, pneumonia induced by SARS‐CoV‐2 infection can manifest with different clinical presentations ranging from mild to severe disease with differing pathological conditions. Therefore, Tregs dose, adjunct therapies, and antigen specificity required for SARS‐CoV‐2 infection pneumonia need to be addressed. For mild patients, for example, a combination of nTregs with immunosuppressive drugs would be the most prudent method. In nonsevere patients, manifestations treatment with less than 0.1–3 × 10^6^ Tregs/kg would seem appropriate for initial treatment.^15^ On the other hand, for more severe patients, antigen‐specific Treg or antigen‐specific iTregs might be considered. Since the precise mechanism(s) of pathological processes associated with SARS‐CoV‐2 infection are still unclear, the use of dosage needs to be carefully considered according to the results of ongoing studies. Indeed, some concerns were noted on the inability of Tregs to mount an antiviral response.^44^, ^53^ Since Treg cells are unlikely to produce off‐target effects, they can be engineered to be “educated” to be able to be controlled as needed to enhance suppressive function and mediate regeneration and tissue repair. Thus, we believe that manipulation of Tregs should be considered for clinical trials to evaluate their effectiveness in improving clinical outcomes and reducing mortality rates of patients infected with COVID‐19.

## 3 CONCLUSIONS

Current pathology reports have revealed that the lung tissues in people infected with SARS‐CoV‐2 were prominently infiltrated with multinucleated giant cells and inflammatory cells. The clinical exacerbation of patients infected with SARS‐CoV‐2 may have resulted from an interaction of cytopathic effects directly caused by virus as well as from immune stimulation known as cytokine‐storm. Tregs cell therapy is widely used to treat various autoimmune and inflammatory diseases in animal models and some clinical trials are going on. Rigorously designed clinical trials and detailed Treg manufacturing planning should be considered for evaluation in clinical trials to evaluate their effectiveness in improving clinical outcomes and reducing mortality rates of patients with COVID‐19.

## CONFLICT OF INTEREST

The authors declare no conflict of interest.
